# *Ulk4* regulates GABAergic signaling and anxiety-related behavior

**DOI:** 10.1038/s41398-017-0091-5

**Published:** 2018-02-02

**Authors:** Min Liu, Marie Fitzgibbon, Yanqin Wang, Jamie Reilly, Xiaohong Qian, Timothy O’Brien, Steve Clapcote, Sanbing Shen, Michelle Roche

**Affiliations:** 1 Regenerative Medicine Institute, School of Medicine, National University of Ireland Galway, Galway, Ireland; 2 Physiology, School of Medicine, Galway Neuroscience Centre and Centre for Pain Research, National University of Ireland Galway, Galway, Ireland; 3 Department of Physiology, College of Life Science, Hebei Normal University, Shijiazhuang, China; 4 National Center for Protein Sciences, Beijing Proteome Research Center, National Engineering Research Center for Protein Drugs, Beijing Institute of Radiation Medicine, Beijing, China; 5 School of Biomedical Sciences, University of Leeds, Leeds, UK

## Abstract

Excitation/inhibition imbalance has been proposed as a fundamental mechanism in the pathogenesis of neuropsychiatric and neurodevelopmental disorders, in which copy number variations of the *Unc-51 like kinase 4* (*ULK4*) gene encoding a putative Serine/Threonine kinase have been reported in approximately 1/1000 of patients suffering pleiotropic clinical conditions of schizophrenia, depression, autistic spectrum disorder (ASD), developmental delay, language delay, intellectual disability, or behavioral disorder. The current study characterized behavior of heterozygous *Ulk4*^*+/tm1a*^ mice, demonstrating that *Ulk4*^*+/tm1a*^ mice displayed no schizophrenia-like behavior in acoustic startle reactivity and prepulse inhibition tests or depressive-like behavior in the Porsolt swim or tail suspension tests. However, *Ulk4*^*+/tm1a*^ mice exhibited an anxiety-like behavioral phenotype in several tests. Previously identified hypo-anxious (*Atp1a2*, *Ptn*, and *Mdk*) and hyper-anxious (*Gria1*, *Syngap1*, and *Npy2r*) genes were found to be dysregulated accordingly in *Ulk4* mutants. Ulk4 was found to be expressed in GABAergic neurons and the Gad67^+^ interneurons were significantly reduced in the hippocampus and basolateral amygdala of *Ulk4*^*+/tm1a*^ mice. Transcriptome analyses revealed a marked reduction of GABAergic neuronal subtypes, including *Pvalb*, *Sst*, *Cck*, *Npy*, and *Nos3*, as well as significant upregulation of GABA receptors, including *Gabra1*, *Gabra3*, *Gabra4, Gabra5*, and *Gabrb3*. This is the first evidence that Ulk4 plays a major role in regulating GABAergic signaling and anxiety-like behavior, which may have implications for the development of novel anxiolytic treatments.

## Introduction

Anxiety disorders are a category of mental disorders, including generalized anxiety disorder, panic disorder and phobias, characterized by feelings of anxiety and fear. They are the most common mental illness with an estimated prevalence of approximately 20% of world population, affecting 69 million of people in the EU^1^. Recently a meta-analysis was carried out and showed that deletions of 33 hypo-anxious genes were accompanied with increased anxiety, whereas deletions of other 34 hyper-anxious genes were shown to result in decreased anxiety, with both presynaptic and postsynaptic genes involved^2^.

Clinical evidence suggests that altered GABA transmission contributes to the pathophysiology of anxiety disorders in humans. The inhibitory neurotransmitter GABA is synthesized from glutamate by two distinct enzymes, Gad67 (encoded by *Gad1*) and Gad65 (encoded by *Gad2*)^3,4^. The levels of circulating GABA in the central nervous system is largely determined by the Gad67^+^ cells, as deletion of the *Gad1* gene results in >90% reduction in basal GABA levels, whereas *Gad2*^*−/−*^ mice expressed normal levels of GABA^5,6^.

Previous studies from our group have shown that *Unc-51 like kinase 4* (*ULK4*), a gene encoding a putative Ser/Thr kinase, is co-expressed in Gad67^+^ neurons in the cortex and hippocampus of mouse and human brains^7^. In a follow-up study, we showed that *ULK4* was deleted in 1.2/1000 of patients with developmental delay, language delay, severe intellectual disability, and behavior disorder^8^. Recent studies from our group and others have demonstrated that Ulk4 is involved in cortical development^8–10^, ciliogenesis, and CSF flow^11^ in the brain.

In this study, we performed a series of behavioral tests in the *Ulk4*^*+/tm1a*^ mice, as patients carry one copy of *ULK4* gene deletion, and the majority of the *Ulk4*^*tm1a/tm1a*^ mutant mice die within the first 3 postnatal weeks^11^. We demonstrated that the *Ulk4*^*+/tm1a*^ mice exhibited anxiety-related phenotype, with no significant alteration in schizophrenia-related behavior such as prepulse inhibition (PPI), acoustic startle reactivity (ASR), or depression-like behavior of Porsolt swim test (PST) and tail suspension test (TST). This anxiety-related phenotype was accompanied by an imbalanced expression of hypo-anxious and hyper-anxious genes, reduced expression of markers for GABAergic interneuron subtypes, decreased Gad67^+^ cells in the hippocampus and basolateral amygdala and increased GABA receptors.

## Materials and methods

### *Ulk4* hypomorph mice

The *Ulk4*^*+/tm1a*^ mice were created from ES cell clone EPD0182_4_E12 on C57BL/6N background^12^. The gene targeting was done by inserting a *FRT-En2SA-IRES-LacZ-PA-hBactP-Neo-PA-FRT-loxP* cassette into the intron 6 of the *Ulk4* gene. The construct was designed to truncate the *Ulk4* transcription after exon 6 by fusing with *En2SA-IRES-LacZ-PA* containing an *En2* splicing acceptance (En2SA) site. We previously shown that low levels of *Ulk4* mRNA expression were detected in homozygous *Ulk4*^*tm1a/tm1a*^ mutants using primers downstream of the exon 6, and this could be resulted from the leaking of the construct and/or alternative splicing, despite the *Ulk4* isoforms are largely unknown. The *Ulk4*^*tm1a/tm1a*^ strain is therefore regarded as a hypomorph model, rather than a null mutant^11^. The genotyping was carried out by polymerase chain reaction (PCR). The wild-type (WT) allele was detected by a 271 bp PCR product using primers Ulk4EndE7For (5′-TAACTTGCTGGACGGATTGCTG-3′) and Ulk4EndIn7Rev (5′-TGATCTGTAATCGCAGTGCAGG-3′). The mutant allele gave rise to a 621 bp DNA band using primers Ulk4KOMPKOFor (5′-GAGATGGCGCAACGCAATTAATG-3′) and Ulk4KOMPKORev (5′-CTGAGGAGACAATGTAACCAGC-3′)^8,11^.

All experimental procedures were approved by the Irish Department of Health and Children in accordance with Cruelty to Animals Act of 1876 and by the Institutional Animal Care and Research Ethics Committee. The *Ulk4*^*+/tm1a*^ and WT littermates were generated from *Ulk4*^*+/+*^ × *Ulk4*^*+/tm1a*^ mating for behavioral tests and immunohistochemistry. Mice were group-housed according to gender after weaning, and kept at 21 ± 2 °C under 12:12 h light dark (lights on from 07.00 to 19.00 h). All behavioral experiments were carried out between 10.00 and 17.00 h from 8 week old. For RNA sequencing, the *Ulk4*^*tm1a/tm1a*^ mice and WT littermates were produced by *Ulk4*^*+/tm1a*^ × *Ulk4*^*+/tm1a*^ mating.

#### Three-chamber sociability and social novelty preference test

The sociability and social novelty preference test were carried out in a three-chamber apparatus^13–16^. Each animal was placed into the center of the arena and allowed access to all chambers for 10 min. Distance moved and time spent in each compartment was assessed to evaluate general locomotor activity and preference for any compartment. Following this acclimatization period, two identical small cages were placed in the two-side chambers, one containing an unfamiliar stimulus mouse of the same sex, which was randomly assigned to either the right or left chamber of the arena (sociability testing). The test animal was then allowed to freely explore the entire arena for a further 10 min and behavior recorded. Following this, the test animal was confined to the central chamber, while a second unfamiliar mouse of the same sex to the test mouse was placed into the empty wire cage (social novelty preference). The test animal was then allowed to freely explore the arena for further 10 min and behavior recorded. Distance moved in the arena, time spent in each chamber, as well as duration and frequency of engaging in investigatory behavior were assessed. All behaviors were evaluated with the aid of EthoVision XT software (Noldus Netherlands).

#### Novel open field test

Mice were placed in a novel open field arena (30 × 30 × 30 cm) for a 20-min period and behavior was recorded onto a DVD. Distance moved and time spent on grooming were assessed using Ethovision XT software (Noldus Netherlands).

#### Marble burying test (MBT)

The MBT is a well-validated screen for anxiety/neophobia-related behavior. This test was carried out as described^17,18^ with minor modifications. A novel test cage contained 15-cm deep wood shavings bedding, with 15 black glass marbles arranged in an equidistant 5 × 3 grid on top of the bedding. Animals were placed in the testing arena for 20 min, and the number of buried marbles was recorded and statistically analyzed.

#### Elevated plus maze (EPM)

The EPM arena consisted of a wooden apparatus, elevated to a height of 55 cm above the floor, with two open (50 × 10 cm, lux 65) and two closed arms (50 × 10 × 30 cm, lux 30) extending from a central platform (10 × 10 cm)^19^. Mice were placed individually on the central platform facing an open arm and behavior in the arena recorded over a 5-min period. Anxiety-related behavior was analyzed by using Ethovision XT software and the time spent in open and closed arms, and the number of entries by the subjects into the open arms assessed. Locomotor activity was assessed as distance moved over the trial duration.

#### Histology and immunohistochemistry

The histology and immunohistochemistry were carried out as previously described^8,11^ in five WT females and five *lk4*^*+/tm1a*^ females. The primary antibody was mouse anti-Gad67 (MAB5406, Millipore). The secondary antibody was biotinylated goat anti-mouse (71-00-18, KPL). Gad67^+^ neurons were stained with diaminobenzidine, and imaged under a bright filed microscope (IX41, Olympus) equipped with a camera.

Brain structures were delineated according to the mouse brain atlas^20^. Images were taken at 4 × objective lens, under an Axiovert 40CFL microscope (Zeiss). Cell counting for GAD67^+^ neurons was performed on the basolateral amygdaloid complex surrounded by the amygdala capsule (Bregma-1.34–1.70 mm). The mean cell numbers in the outlined basolateral amygdala and hippocampus from 6–10 coronal serial sections/mouse were used for statistical analysis. The X-gal staining was carried out to reveal the Ulk4 expression pattern as the *Ulk4*^*+/tm1a*^ strain was an insertional mutation. The staining solution contained 1 mg/ml X-gal, 5 mm K4Fe(CN)6, 5 mm K3Fe(CN)6, 2 mm MgCl2, 0.02% Nonidet P-40, 0.01% sodium deoxycholate in PBS^11^.

### RNA transcriptome analyses and target validation

RNA was extracted from P12 cortex of one male and two female WT mice and three female *Ulk4*^*tm1a/tm1a*^ mutants. Whole-genome RNA sequencing and analyses were performed by BGI and transcriptome was analyzed on the top 1945 differentiated expressed genes as previously described^11^. Quantitative RT-PCR was carried out to validate gene expression of selected Ulk4 targets. Single-strand cDNA was reversely transcribed from cortical RNA of four WT and four *Ulk4*^*tm1a/tm1a*^ mice. Triple qRT-PCR reactions were performed for each primer pair, included PvalbFor 5′-TGTCGATGACAGACGTGCTC-3′ and PvalbRev 5′-ACCTTCTTCACCTCATCCGG-3′ (138 bp); MdkFor 5′-TGAAGAAGGCGCGGTACAATG-3′ and MdkRev 5′-ATATCTTGGGCCTGTGGGAGA-3′ (186 bp); Gng2For 5′-GATAAGCTGTCGCCCCATGT-3′ and Gng2Rev 5′-GGGTTCATTCCTGCTTGGAG-3′ (158 bp); Slc38a1For 5′-GCCATGTCTTGGTGACCATC-3′ and Slc38a1Rev 5′-ATCTCCGTCCTGGTTCGTGA-3′ (167 bp); Slc38a2For 5′-TTACGGACACGTGGAATCGG-3′ and Slc38a2Rev 5′-AGTGACGGAACTCCGGATAG-3′ (148 bp); Slc38a3For 5′-TGTTCTGTTCCCGGTACGAC-3′ and Llc38a3Rev 5′-GTTGGGGGCGAAGATAACCA -3′ (133 bp). Each plate was loaded with house-keeping reaction mixtures and corrected for GapdhFor 5′-CTCATGACCACAGTCCATGC-3′ and GapdhRev 5′-CACATTGGGGGTAGGAACAC-3′ as cDNA loading. Relative RNA abundance in the *Ulk4*^*tm1a/tm1a*^ cortex was calculated using 2^ΔΔCt^, with average expression level of the corresponding gene in the WT littermates as 100%. The data were presented as mean ± standard error of the mean (SEM), *n* = 4 each, *p < 0.05.

### Statistical analysis

All data were expressed as mean ± SEM. Behavioral data were analyzed with two-way ANOVA when both sex and genotype were considered or one-way ANOVA when only one factor was considered. The immunohistochemistry data were analyzed using one-way ANOVA. Data were considered significant when *p* < 0.05. For RNA sequencing, expression data were normalized to FPKM (fragments per kilobase of transcript per million mapped reads). The differentially expressed genes were shortlisted with *p*-values of multiple testing and false discovery rates (FDRs), and transcriptome pathways were carried out as previously analyzed^11^.

## Results

### *Ulk4*^*+/tm1a*^ male mice exhibit impaired social novelty preference

*Ulk4*^*+/tm1a*^ mice were comprehensively examined for behavioral changes, as single-copy deletion of the *ULK4* gene was identified in patients of neuropsychiatric and neurodevelopmental disorders^7,8^. We tested schizophrenia-related positive or negative symptoms by ASR, PPI, and depression-related behavior using the PST and TST. *Ulk4*^*+/tm1a*^ mice exhibited no significant difference from the WT littermates in any of these tests (Supplementary Figs. S1, S2, S3).

**Fig. 1:**
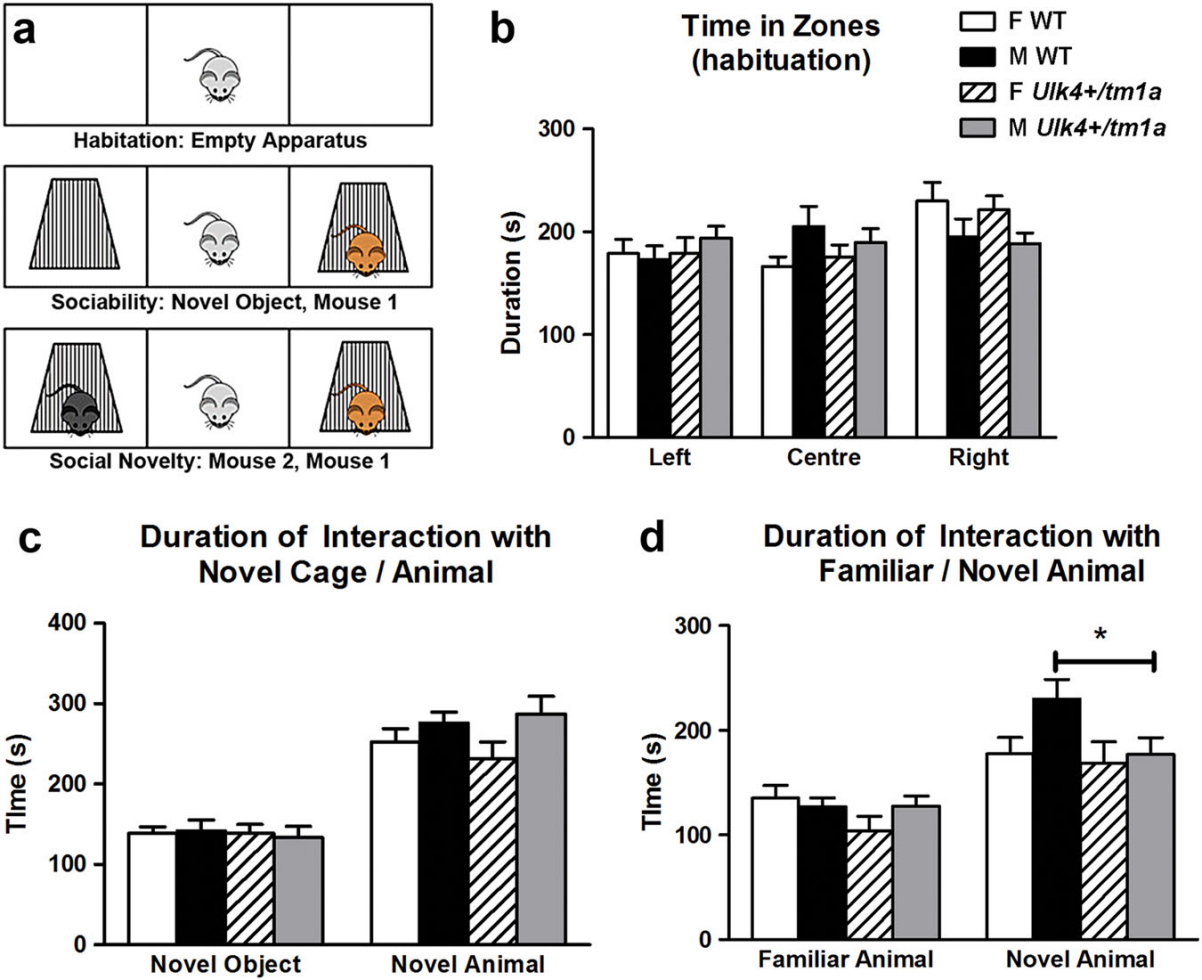
*Ulk4*^*+/tm1a*^ male mice display reduced social novelty preference. **a** WT male (*n* = 18), WT female (*n* = 20), *Ulk4*^*+/tm1a*^ male (*n* = 20), and *Ulk4*^*+/tm1a*^ female (*n* = 14) were tested in three-chamber apparatus. **b** No zone preference during the habitation period (*p* > 0.05). **c** No genotype difference in sociability test. All animal spent more time in the arena with the novel stimulus animal vs. time in center or in arena of novel object (*p* < 0.01). **d** Social novelty tests showing that *Ulk4*^*+/tm1a*^ male spent significantly less time interacting with the novel animal in comparison to the WT male (*p < 0.05)

We next investigated the sociability and preference for social novelty in three-chamber apparatus (Fig. 1a), as impaired social behavior is common in neurodevelopmental and neuropsychiatric disorders. During the 10-min habitation period, *Ulk4*^*+/tm1a*^ mice displayed no zone preference (Fig. 1b). In the subsequent 10-min sociability test, all animals spent significantly (*p* < 0.01) more time in the chamber with a novel con-specific mouse (of the same sex as the test mouse) than in the center zone or the chamber with an empty cage, with no statistical difference between WT and *Ulk4*^*+/tm1a*^ mice (Fig. 1c). In the social novelty preference test (Fig. 1d), all mice spent significantly increased time in the chamber with a novel mouse than the chamber containing the familiar mouse (*p* < 0.01). However, *Ulk4*^*+/tm1a*^ male spent significantly less time interacting with the novel male mouse in comparison to WT male (Fig. 1d, *p* < 0.05), an effect not observed in female counterparts. This suggests that *Ulk4* mutation may influence social novelty preference of male mice.

### *Ulk4*^*+/tm1a*^ female mice exhibit anxiety-related behavior in the MBT

**Fig. 2:**
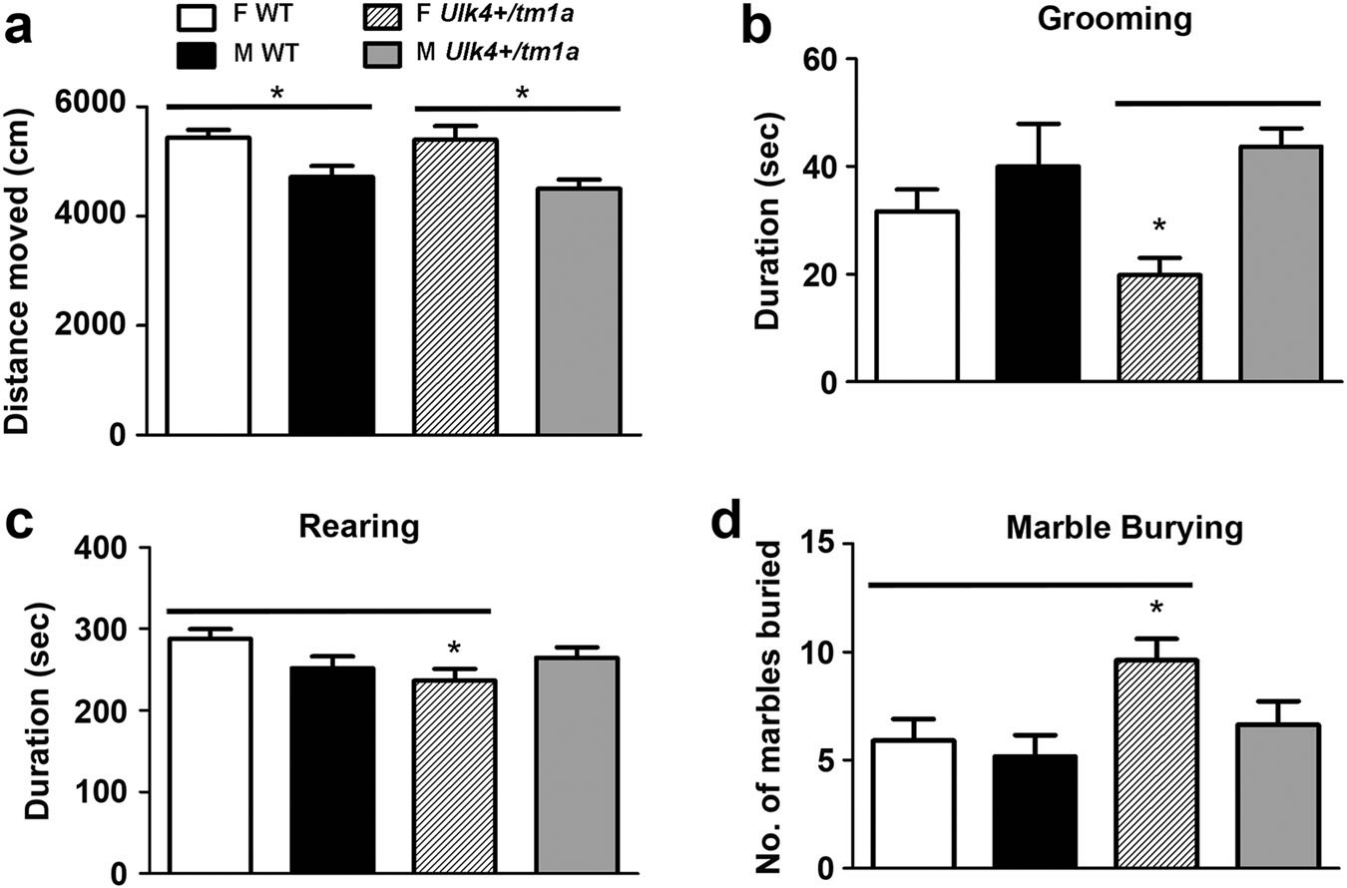
*Ulk4*^*+/tm1a*^ female mice displayed decreased grooming activity, reduced rearing, but increased marble burying. **a**–**c** 20 WT female, 18 WT male, 13 *Ulk4*^*+/tm1a*^ female, and 17 *Ulk4*^*+/tm1a*^ male at 2–3-month-old were placed in an open field arena for 20 min. **a** There was no genotype-specific but gender-associated difference in distance traveled. **b** The duration spent on grooming was reduced in *Ulk4*^*+/tm1a*^ female (39.8 + 5.1, *n* = 13) compared with *Ulk4*^*+/tm1a*^ male (77.3 + 6.3, *n* = 17, *p* < 0.01). **c** *Ulk4*^*+/tm1a*^ female also showed reduced rearing activity (240.2 + 14.2, *n* = 13, *p* = 0.02) than WT Female (287.7 + 12.1, *n* = 20). **d** *Ulk4*^+/*tm1a*^ female and total *Ulk4*^*+/tm1a*^ mice showed significant increase in marble burying compared to WT female and total WT mice

Repetitive or stereotypical behavior is common in anxiety and ASD. As such we examined duration of grooming behavior as a measure of repetitive activity in open field test. There was no genotype-specific difference on the distance moved during the test (*p* = 0.23), although males (both WT and *Ulk4*^*+/tm1a*^*)* displayed lower locomotor activity than corresponding females (Fig. 2a). For grooming behavior, *Ulk4*^*+/tm1a*^ male mice displayed more repetition-like activity, with grooming time doubled (77.3 ± 6.3 s, *n* = 17) when compared to *Ulk4*^*+/tm1a*^ female (39.8 ± 5.1 s, *n* = 13, *p* < 0.01, Fig. 2b), whereas WT male mice exhibited no difference in duration of grooming from WT female (*p* = 0.20). Temporal analysis of grooming behavior over the 20 min trail demonstrated that *Ulk4*^*+/tm1a*^ female failed to increase grooming activity in the last 10 min of the trial when compared to other groups (WT female: 31.63 ± 4.10 s vs. *Ulk4*^*+/tm1a*^ female: 19.86 ± 3.18 s; *p* < 0.05), and also showed lower rearing activity than WT female (Fig. 2c), suggesting that *Ulk4*^*+/tm1a*^ females fail to acclimatize the novel arena possibly due to enhanced anxiety-related behavior.

To further evaluate anxiety-like behavior, we carried out the MBT^14,15^. *Ulk4*^*+/tm1a*^ females buried significantly more marbles (9.6 ± 1.0, *n* = 13) than WT females (5.9 ± 1.0, *n* = 20, *p* = 0.01, Fig. 2d). The *Ulk4*^*+/tm1a*^ group as a whole also buried significantly more marbles (7.8 ± 0.8, *n* = 32) than the WT group (5.6 ± 0.7, *n* = 38, *p* = 0.03). This further confirmed anxiety-like or neophobia-like behavior in *Ulk4*^+/*tm1a*^ mice.

### *Ulk4*^*+/tm1a*^ mice exhibit anxiety-related behavior on the EPM

**Fig. 3:**
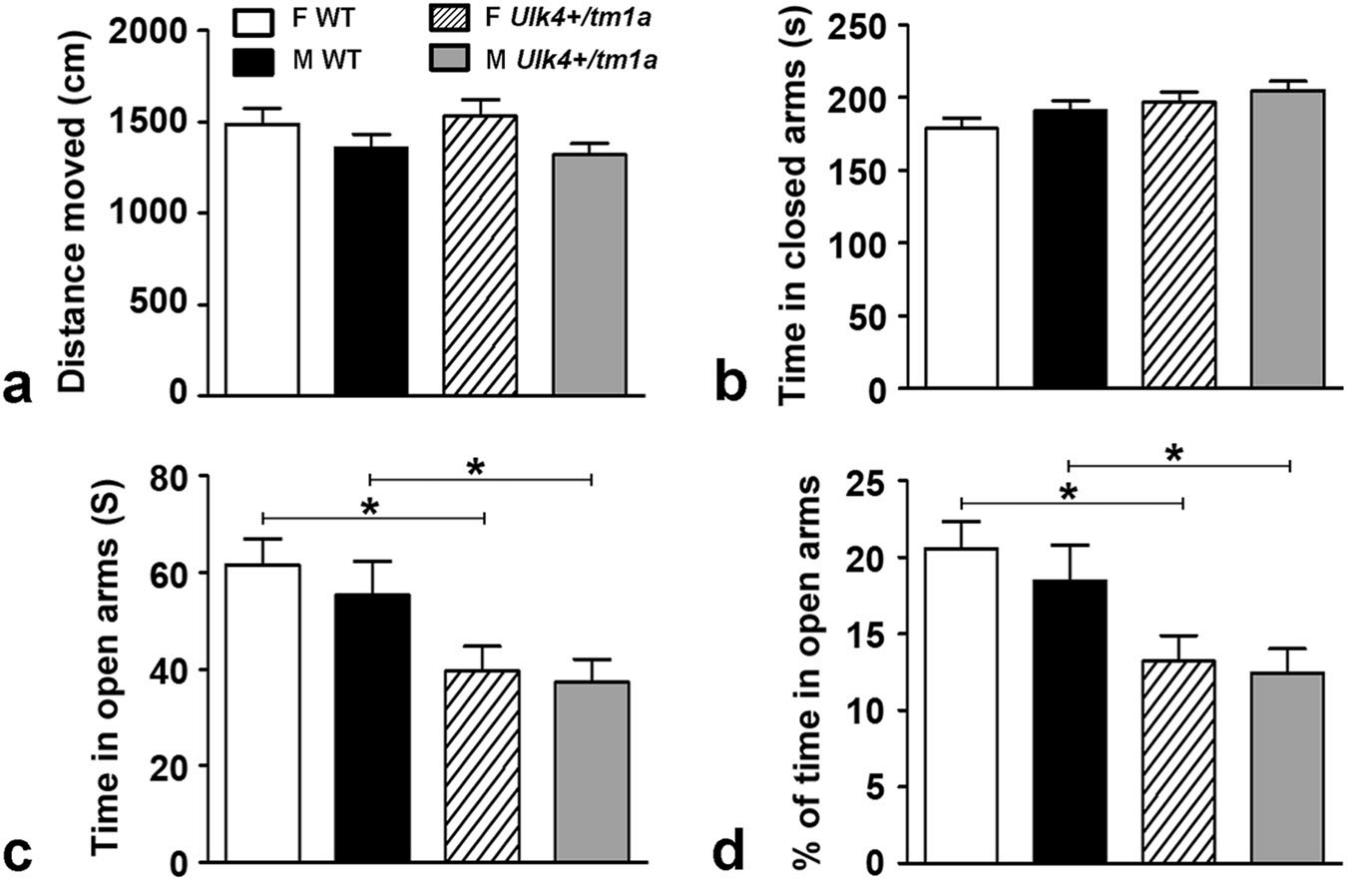
Both *Ulk4*^*+/tm1a*^ male and female mice exhibit an increased anxiety on EPM test. **a** Distance moved; **b** time spent in the closed arms; **c** time spent in open arms; and **d** percentage of time spent in the open arms. Male and female *Ulk4*^*+/tm1a*^ mice and their WT littermates were subjected to the EPM. Data are expressed as mean ± SEM; *n* = 12–19 per group. *p < 0.05

We next carried out EPM test^16^ to further assess anxiety-like behavior in the mutant mice. Female mice exhibited greater locomotor activity on the EPM than males in consistency with the open field test, although this was not genotype-specific (Fig. 3a). Both *Ulk4*^*+/tm1a*^ male and female mice spent significantly less time in the open arms of the EPM than WT counterparts (Fig. 3c, d). Thus, *Ulk4*^*+/tm1a*^ mice display an elevated anxiety-like behavior in the EPM.

### *Ulk4*^*+/tm1a*^ mice show fewer Gad67^+^ cells in basolateral amygdala and hippocampus

**Fig. 4:**
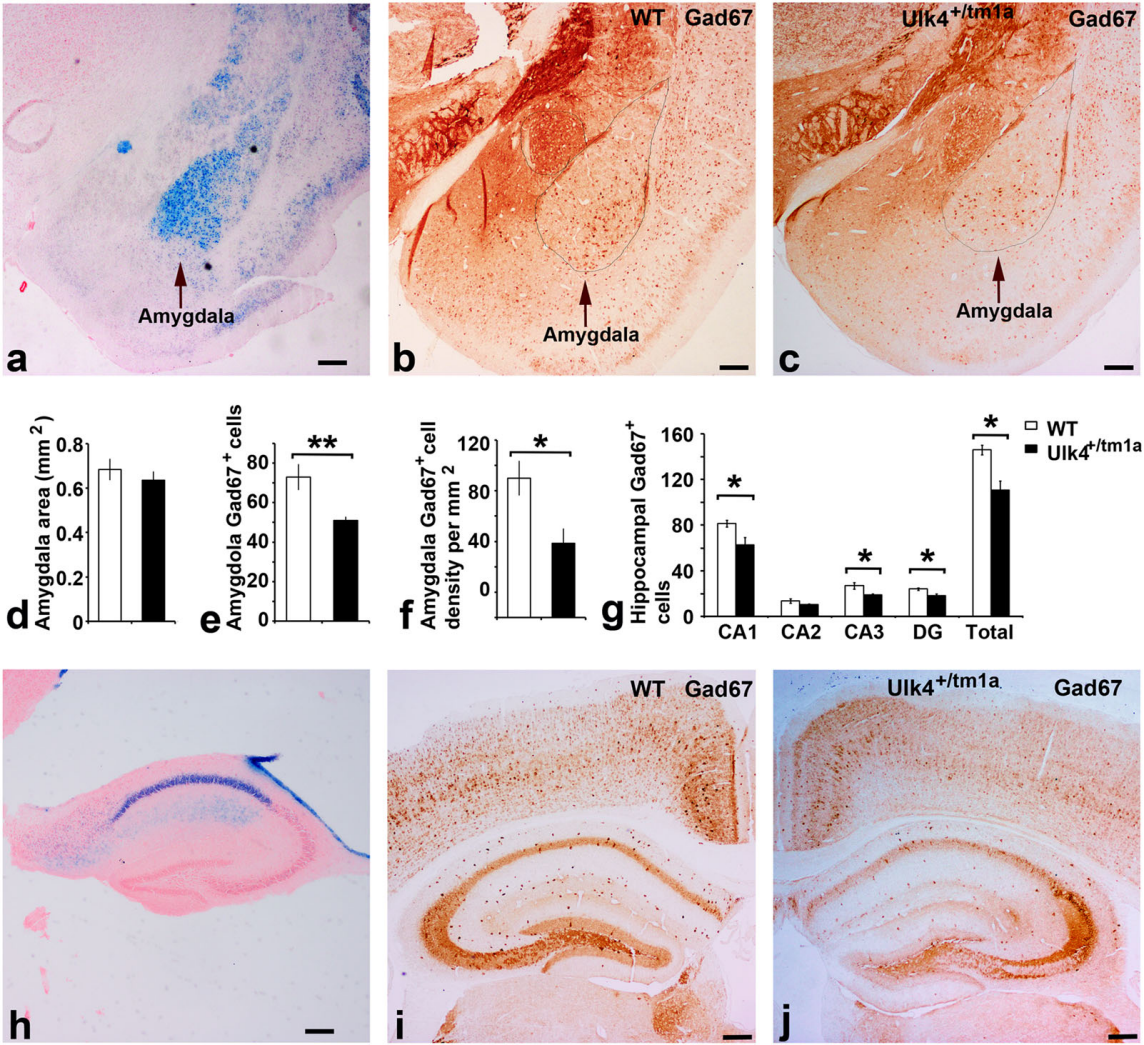
Reduced Gad67^+^ cells in the amygdala and hippocampus of *Ulk4*^*+/tm1a*^ mice. X-gal staining showed Ulk4 expression in the basolateral amygdala (**a**) and hippocampus (**b**). Anti-Gad67 immunohistochemistry was carried out on brain section of five female WT and five *Ulk4*^*+/tm1a*^ female mice at 2 months. The Gad67^+^ cells were quantified from the equivalent regions as outlined in WT (0.68 ± 0.05 mm^2^, *n* = 5) and *Ulk4*^*+/tm1a*^ (0.64 ± 0.04 mm^2^, *n* = 5) mice. The Gad67^+^ cells were quantified from comparable sections of the CA1 (81.2 ± 3.0 in WT vs. 62.9 ± 6.1 in *Ulk4*^*+/tm1a*^, *p* = 0.03), CA2 (13.7 ± 1.9 in WT vs. 10.3 ± 0.7 in *Ulk4*^*+/tm1a*^, *p* = 0.13), CA3 (27.0 ± 2.8 in WT vs. 19.0 ± 0.9 in *Ulk4*^*+/tm1a*^, *p* < 0.02), DG (24.1 ± 1.2 vs. 18.4 ± 1.4, *p* < 0.02), WT hippocampus (146.0 ± 4.3) and *Ulk4*^*+/tm1a*^ hippocampus (110.6 ± 8.2, *p* < 0.01) and statistically analyzed (**g**), showing significant reduction of Gad67 cells (**e**) and cell density in *Ulk4*^*+/tm1a*^ Amygdala (**f**) and a significant reduction (*p* < 0.05) of Gad67 cells in the hippocampus (**g**) of *Ulk4*^*+/tm1a*^ mice. Bar = 200 µm in **a**, **b**, **c**, **h**, **i**, **j**. *p < 0.05; **p < 0.01

Neuroanatomic analysis demonstrated normal brain morphology in *Ulk4*^*+/tm1a*^ mice, with no schizophrenia-related or hydrocephalus-related phenotype (Supplementary Fig. 4). Thus, a single copy of *Ulk4* gene deletion does not affect gross neuroanatomy in mice. We previously showed that Ulk4 is expressed in GABA neurons^7^, and altered GABA signaling is implicated in neuropathology of the neuropsychiatric illness. The amygdala and hippocampus are key brain regions associated with anxiety, fear, and learning. We took advantage of *lacZ* reporter gene in the Ulk4 mutant, performed X-gal staining, and demonstrated the strongest β-gal activity in the medial part of the basolateral amygdala (Fig. 4a) and in the CA1 region of the hippocampus (Fig. 4h). We subsequently carried out anti-Gad67 staining in 2-month WT and *Ulk4*^*+/tm1a*^ mice, and quantified Gad67^+^ cells (Fig. 4b, c) from comparable sections in the basolateral amygdaloid complex and hippocampus. The data revealed there were 72.9 ± 6.3 Gad67^+^ cells in the WT basolateral amygdala, with a cell density of 109.8 ± 13.3 cells/mm^2^ (Fig. 4b, c). In *Ulk4*^*+/tm1a*^ mice, 50.9 ± 1.7 Gad67^+^ cells were found in the equivalent region with a cell density of 58.7 ± 11.1 (Fig. 4b–e). These data demonstrated a significant reduction of Gad67^+^ cells (*p* = 0.01) and cell density (*p* = 0.02) in the amygdala of the *Ulk4*^*+/tm1a*^ mice.

In the hippocampus, an average of 146.0 ± 4.3 Gad67^+^ cells was found on the WT hippocampus, whereas *Ulk4*^*+/tm1a*^ mice exhibited an average cell number of 110.6 ± 8.2 cells (Fig. 4f, g, i, j, *n* = 5 each, *p* < 0.01). This was resulted from general decrease of Gad67^+^ cells in the CA1 (81.2 ± 3.0 vs. 62.9 ± 6.1, *p* < 0.05), CA2 (13.7 ± 1.9 vs. 10.3 ± 0.7, *p* > 0.05), CA3 (27.0 ± 2.8 vs. 19.0 ± 0.9, *p* < 0.05), and DG (24.1 ± 1.2 vs. 18.4 ± 1.4, *p* < 0.05) of *Ulk4*^*+/tm1a*^ vs. WT mice. Therefore, these data demonstrated that *Ulk4*^*+/tm1a*^ mice exhibit reduced numbers of GABAergic neurons in the basolateral amygdala and hippocampus, thus possibly provide the cellular mechanism for the anxiety-like behavior in *Ulk4*^*+/tm1a*^ mice.

### Reduced expression of markers for GABAergic interneuron subtypes in the *Ulk4*^*tm1a/tm1a*^

GABAergic interneurons are the major inhibitory neurons, with >12 distinct subtypes of GABAergic interneurons expressing calretinin (Calb2), somatostatin (Sst), cholecystokinin (Cck), neuropeptide Y (Npy), vasointestinal polypeptide (Vip), choline acetyltransferase (Chat), and NOS^21^. To systematically evaluate the effect of *Ulk4* lesion of the various GABA interneuron subtypes, we carried out transcriptome analyses in the WT and *Ulk4*^*tm1a/tm1a*^ mice by whole-genome RNA sequencing. The expression of *Pvalb*, *Sst*, *Cck*, *Npy*, and *Nos3* were all reduced in *Ulk4* mutants (Table 1). Pvalb-expressing cells constitute 39% of GABA interneurons and are vital for neuronal synchronization. Notably *Pvalb* was 37% reduced in *Ulk4* mutants (*p* = 4.33E-07, FDR = 9.01E-06). Together these data support a substantial reduction of pre-synaptic GABA interneurons in *Ulk4* mutants.

**Table 1:** Ulk4 regulates GABAergic signaling, hyper-anxious, and hypo-anxious genes

	WT (FPKM)	*Ulk4* mutants (FPKM)	Average mutant/WT	Up-down (mutant/WT)	*p*-value	FDR
GABA subtypes						
*Pvalb* (39%)	28.7 ± 2.9	18.0 ± 3.0	0.63	Down	4.33E-07	9.01E-06
*Sst* (23%)	382.4 ± 22.8	324.9 ± 11.9	0.85	Down	7.97E-10	2.61E-08
*Cck* (5%)	437.7 ± 3.8	358.0 ± 1.9	0.82	Down	2.52E-17	1.80E-15
*Npy* (8%)	257.1 ± 9.9	196.8 ± 21.4	0.77	Down	1.19E-14	6.84E-13
*Nos3* (<1%)	4.6 ± 0.4	3.1 ± 0.6	0.67	Down	1.09E-04	1.35E-03
*Calb2* (24%)	11.2 ± 0.3	16.3 ± 1.8	1.45	Up	5.29E-05	7.07E-04
*Vip* (11%)	18.9 ± 1.3	23.2 ± 3.4	1.23	Up	4.04E-03	3.05E-02
GABAergic synapse pathway						
*Gng4*	47 ± 3.1	40.1 ± 4.3	0.85	Down	5.26E-05	7.04E-04
*Gng7*	142.6 ± 15.9	107.8 ± 33.4	0.76	Down	5.00E-53	1.82E-50
*Slc38a3*	42.5 ± 5.6	31.5 ± 7.4	0.74	Down	2.37E-10	8.49E-09
*Slc38a5*	7.1 ± 1.4	3.7 ± 1.4	0.53	Down	8.81E-06	1.43E-04
*Adcy9*	9.7 ± 0.8	13.4 ± 2.2	1.39	Up	1.91E-13	9.77E-12
*Cacna1c*	7.2 ± 0.4	8.6 ± 0.5	1.19	Up	3.57E-05	4.98E-04
*Gabra1*	78.4 ± 8.5	95 ± 8.7	1.21	Up	2.55E-22	2.60E-20
*Gabra3*	36.5 ± 2.5	44.4 ± 3	1.21	Up	1.24E-08	3.39E-07
*Gabra4*	32.7 ± 1.6	38.8 ± 1.5	1.19	Up	3.91E-07	8.21E-06
*Gabra5*	35.5 ± 0.3	43.3 ± 4.9	1.22	Up	2.03E-06	3.76E-05
*Gabrb3*	74.8 ± 5.8	95.5 ± 8.9	1.28	Up	6.62E-35	1.29E-32
*Gng2*	85.6 ± 2	103.2 ± 10.7	1.20	Up	1.35E-16	8.93E-15
*Hap1*	15.7 ± 2.2	26.6 ± 5	1.70	Up	4.26E-24	4.92E-22
*Nsf*	174.4 ± 15.1	214.9 ± 9.8	1.23	Up	3.11E-38	7.03E-36
*Slc38a1*	21.7 ± 0.8	28.1 ± 1	1.30	Up	5.89E-18	4.41E-16
*Slc38a2*	21.3 ± 0.6	33.6 ± 8.4	1.58	Up	5.01E-33	9.04E-31
Hypo-anxious genes						
*Atp1a2*	228.0 ± 18.7	175.47 ± 19.7	0.77	Down	1.28E-117	1.40E-114
*Ptn*	178.6 ± 26.3	142.9 ± 33.5	0.80	Down	9.28E-19	7.57E-17
*Mdk*	22.8 ± 3.9	14.5 ± 6.1	0.63	Down	1.47E-04	1.75E-03
*Plcb4*	11.4 ± 2.4	14.9 ± 2.5	1.31	Up	1.50E-05	2.28E-04
*Apoe*	1085.4 ± 162.0	1127.2 ± 46.3	1.04	Up	1.56E-05	2.36E-04
*Cacna1e*	15.1 ± 3.5	17.0 ± 0.3	1.13	Up	2.75E-04	3.06E-03
*Adra2a*	8.4 ± 1.1	10.7 ± 1.7	1.28	Up	3.72E-04	3.97E-03
Hyper-anxious genes						
*Ncam1*	96.0 ± 4.7	82.8 ± 8.5	0.86	Down	4.78E-12	2.09E-10
*Gria1*	48.4 ± 6.7	59.4 ± 8.3	1.23	Up	4.14E-16	2.65E-14
*Syngap1*	106.8 ± 10.0	121.9 ± 27.1	1.14	Up	3.03E-10	1.06E-08
*Npy2r*	0.9 ± 0.3	1.9 ± 0.6	2.13	Up	1.15E-04	1.41E-03
*Ptpra*	56.6 ± 2.2	62.9 ± 2.4	1.11	Up	1.98E-04	2.29E-03

### Disrupted GABAergic synapse in *Ulk4*^*tm1a/tm1a*^ mice

Analyses of the top 1945 differentially regulated genes (FDR < 0.025, *p* < 0.0033) identified “Synapse” pathway (FDR = 1.51E-18) with 124 genes significantly altered in *Ulk4* mutants. More “Postsynapse” genes (*n* = 72, FDR = 1.55E-12) were affected than “Presynapse” genes (*n* = 26, FDR = 1.03E-03). KEGG pathway analyses showed that 16 genes in the GABAergic synapse (FDR = 0.016) were significantly dysregulated in *Ulk4* mutants (Table 1). Whereas *Gng7*, *Gng4*, *Slc38a3*, and *Slc38a5* were downregulated in mutants, postsynaptic targets *Gabra1*, *Gabra3*, *Gabra4*, *Gabra5*, and *Gabrb3* genes were significantly upregulated, together with overexpression of *Nsf*, *Slc38a1*, *Slc38a2*, *Hap1*, *Gng2*, *Adcy9*, and *Cacna1c*. This highlights that *Ulk4*^*tm1a/tm1a*^ mutation significantly alters both pre-synaptic and post-synaptic GABAergic signaling.

### *Ulk4* regulates hyper-anxious and hypo-anxious gene expression

The previous meta-analysis identified 33 hypo-anxious and 34 hyper-anxious genes^2^. The quantitative RNA sequencing showed that three hypo-anxious genes *Atp1a2* (FDR = 1.40E-114), *Ptn* (FDR = 7.57E-17), and *Mdk* (FDR = 1.75E-03) were reduced to 77, 80, and 63% in *Ulk4* mutants. Meanwhile, four hyper-anxious genes *Gria1* (123%, FDR = 2.65E-14), *Syngap1* (114%, FDR = 1.06E-08), *Npy2r* (213%, FDR = 0.001), and *Ptpra* (111%, FDR = 0.002) were significantly increased. Therefore deletion of the *Ulk4* gene is associated with imbalanced expression of hyper-anxious and hypo-anxious genes (Table 1).

### Validation of the *Ulk4* target gene expression

**Fig. 5:**
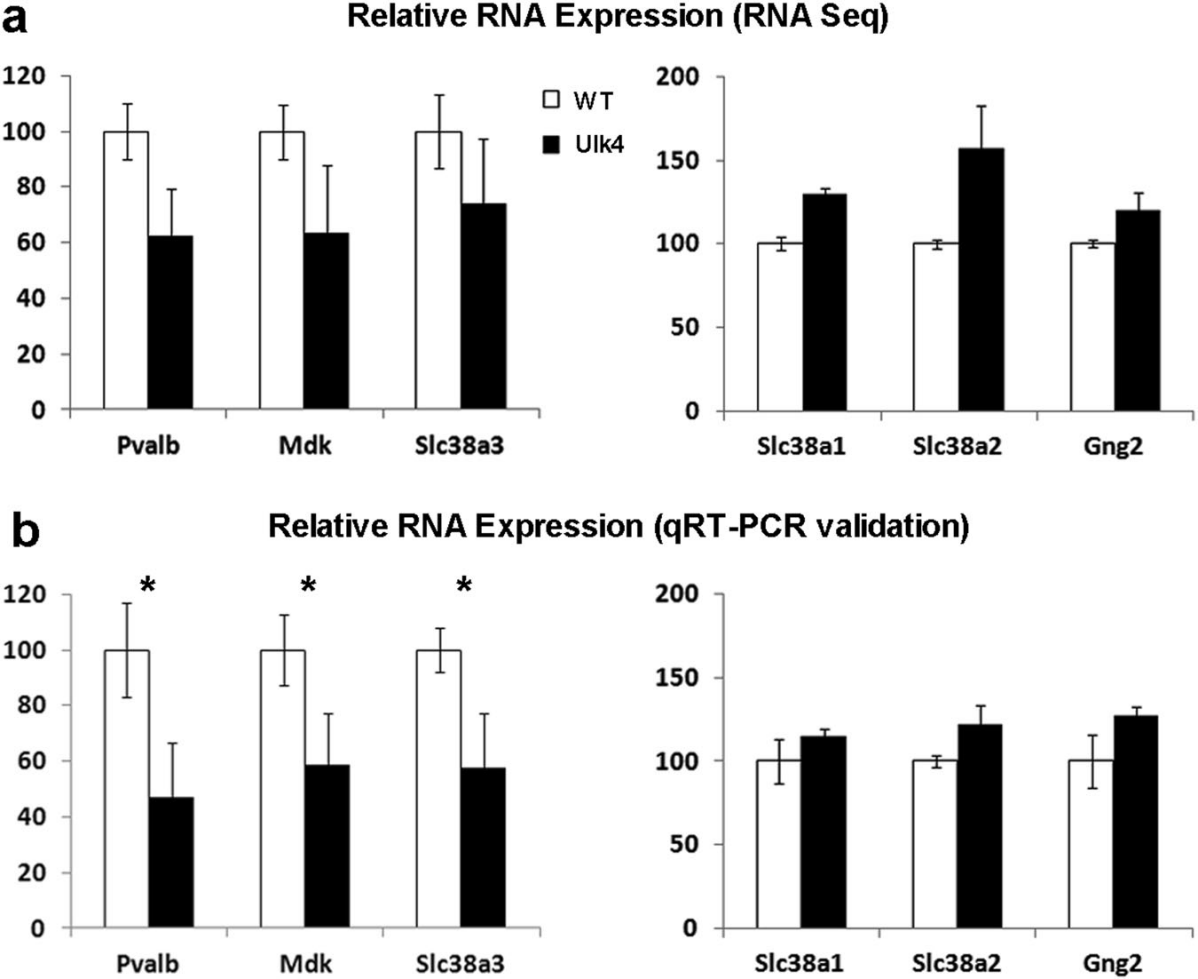
Altered gene expression identified by whole-genome RNA sequencing (a), and validated by quantitative RT-PCR (b). RNA sequencing was carried out in three pairs of P12 WT and *Ulk4*^*tm1a/tm1a*^ cortex, and quantitative RT-PCR in four pairs of cortical RNA (**b**). Whereas *Slc38a1*, *Slc38a2*, and *Gng2* genes showed increased mRNA transcripts by both methods, the *Pvalb*, *Mdk*, and *Slc38a3* gene transcription were significantly reduced (*p < 0.05)

Whole-genome RNA sequencing is one of the most sensitive methods to detect transcriptome changes at the genome level. Here, we carried out quantitative RT-PCR on six selected *Ulk4* targets, the downregulated *Pvalb*, *Mdk*, and *Slc38a3*, and upregulated *Slc38a1*, *Slc38a2*, and *Gng2*. The transcription of *Slc38a1*, *Slc38a*2, and *Gng2* was increased to 115, 122, and 127%, respectively, in the *Ulk4* vs. WT counterparts, but was statistically not significant (Fig. 5, *p* > 0.05). However, the *Pvalb*, *Mdk*, and *Slc38a3* transcripts were reduced to 46.9, 58.7, and 57.8% (Fig. 5, *n* = 4 each, *p* < 0.05), respectively, in the *Ulk4* mutants vs. WT controls. Of particular relevance is the significant reduction of *Pvalb* transcripts for parvalbumin, which marks a major subgroup of GABA inhibitory interneurons. Parvalbumin deficiency is a landmark neuropathology of schizophrenia^22^ and is associated with mouse model of ASD^23^.

## Discussion

Previously, we showed that ULK4 was expressed in GABA neurons and *ULK4* CNVs were found in neurodevelopmental and neuropsychiatric disorders^7,8^. In this study, we report the first evidence that Ulk4 regulates GABAergic signaling in the brain, and *Ulk4* heterozygous mice display anxiety-related phenotype, a prominent component of many neuropsychiatric diseases.

The *ULK4* CNVs occur at a rate of 0.14% (16/11,633) from two previous studies^7,8^, lower than *NRXN1* CNVs as a major target for schizophrenia (0.18–0.63%)^24–26^, and ASD (0.45%)^27,28^. The *ULK4* mutation rate may rise when the whole-exome or genome are sequenced. However, the clinical features of *ULK4* CNVs are pleiotropic, which include schizophrenia, ASD, bipolar disorder^7^, developmental delay, severe language delay, learning difficulties, and behavioral disorder^8^. Therefore, comprehensive behavioral tests were performed on the *Ulk4*^*+/tm1a*^ mice in this study. *Ulk4*^*+/tm1a*^ mice exhibit no obvious schizophrenia-related or depression-related phenotype, as they did not show statistic difference from WT littermates in ASR, PPI, PST, or TST.

However, *Ulk4*^*+/tm1a*^ mice display anxiety-related behavior in a number of behavioral paradigms. Both *Ulk4*^*+/tm1a*^ male and female mice spent significantly less time on the open arms but more time in the closed arms in the EPM test, a well-recognized measure of anxiety-related behavior. *Ulk4*^*+/tm1a*^ male mice interacted less with the novel vs. familiar animal, indicative of a deficit in social novelty preference possibly due to social neo-phobia. *Ulk4*^*+/tm1a*^ females failed to acclimatize the novel open field arena at the end phase of the test, seen as a lack of increase in grooming behavior, and buried significantly more marbles in the MBT. The lack of acclimatization is likely due to the enhanced anxiety of these animals in a novel environment and increased marble burying is a well-recognized behavior associated with neo-phobia/anxiety-related behavior. Thus, both male and female *Ulk4* mutants exhibit anxiety-related behavior in several behavioral paradigms, although some sex-related differences were noted. The reason for sex differences in some but not all of the behavioral tests is unknown; however, Ulk4 may regulate neuro-hormone release and/or activity, as *Ulk4* is expressed also in brain regions mediating homeostatic control, such as the hypothalamus and neurosecretory circumventricular nuclei^29^, which may result in sex-related differences in behavior under certain conditions. Despite the subtle differences in responses between males and females in individual tests, taken together, the behavioral data demonstrate an anxiety-like phenotype in *Ulk4*^*+/tm1a*^ mice.

Consistent with the behavioral changes, three previously identified hypo-anxious genes (*Atp1a2*, *Ptn*, and *Mdk*) were downregulated, and four hyper-anxious genes (*Gria1*, *Syngap1*, *Npy2r*, and *Ptpra*) were significantly upregulated, suggesting that *Ulk4* lesion disturbs balanced expression of hyper-anxious and hypo-anxious genes in the brain^2^. *Ulk4* also plays a role in neurogenesis^8–10^, which is in agreement with the hypothesis that anxious phenotype is associated with neurodevelopmental changes^2^.

In addition, the G-protein-coupled signaling appears also disrupted, as *Gng4*, *Gng7*, *Adcy9*, and *Kcnj6* were dysregulated in *Ulk4* mutants. *Gng4* and *Gng7* are mediators of GPCR signaling linked to fear and anxiety, and *Gng4* expression was inversely correlated with fear and anxiety in the mouse model of post-traumatic stress disorder^30^. *Gng7* was significantly downregulated in the amygdala of *Itpka* KO mice which exhibited fear-related and anxiety-related behaviors^31^. Subtle changes in *Gng7* expression also impacted anxiety and aggressive behaviors in transgenic animals^32^ and in chronic social-defeat stress leading to depressive and anxious states^33^. *ADCY9*, involved in cAMP production, was previously identified as a risk factor for mood disorders^34,35^. However, the relevance of *Kcnj6* (encoding a G-protein activated inward rectifier potassium channel) dysregulation is less certain. *Kcnj6* is located on human Chr21, and trisomy *Kcnj6* transgenic mice display some of the Down syndrome-like neurological abnormalities, but not anxiety-related indices^36^.

Overwhelming evidence suggests that abnormal GABA transmission contributes to the pathophysiology of anxiety disorders in humans^37,38^. For instance, studies using nuclear imaging techniques revealed diminished central GABA and GABA receptor levels in patients with panic disorder, generalized anxiety disorder, and posttraumatic stress disorder^39^. The *GAD2* polymorphisms were shown as a risk factor for anxiety disorders^40^. Genetic variations^41^ and reduced GAD67 expression in bipolar and schizophrenia were also well documented^42,43^. Similarly, *Gad1*^*+/−*^ male mice also exhibit reduced Gad67^+^ neurons, with disturbed characteristics of depression and anxiety^44^. Considering significant decreases in Gad67 neurons in the hippocampus and basolateral amygdala of *Ulk4*^*+/tm1a*^ mice, and the correlating functions of *GAD1* in humans, it will be interesting to investigate if polymorphisms of the *ULK4* link to anxiety disorders in patients.

GABA transmission in the amygdala is considered particularly important in controlling fear and anxiety. We confirmed high Ulk4 expression in the basolateral amygdala and hippocampus using X-gal staining. Anti-Gad67 staining revealed significant reduction of Gad67^+^ neurons in hippocampus and amygdala of the *Ulk4*^*+/tm1a*^ mice. Interestingly, high β-gal activity is located in the medial aspect of the basolateral amygdala, whereas Gad67^+^ cells are more abundant in the lateral aspect of the basolateral amygdala. In the hippocampus, strong X-gal staining is present in the CA1, while Gad67^+^ cells are reduced in all hippocampal regions. These anatomic differences in the patterns of X-gal and Gad67 staining may be due to alternative splicing of the *Ulk4* gene, for example, Ulk4 isoforms may be started after exon 6, which will not be revealed by X-gal staining. Alternatively, Ulk4 protein may also be expressed in other neuronal types, other than Gad67^+^ neurons, such as Gad65^+^ neurons or other neuronal subtypes. We cannot rule out that the change in Gad67^+^ cell number in *Ulk4* mutants may also be an indirect or compensatory consequence of *Ulk4* deletion during development and not be related to loss of Ulk4 function in GABAergic neurons. Regardless of the precise mechanism, the data herein demonstrate that *Ulk4*^*+/tm1a*^ mice exhibit reduced cell number of Gad67^+^ neurons and thus possibly altered GABAergic neurotransmission in the BLA and hippocampus that may underlie the behavioral changes observed.

In humans benzodiazepines are the most widely prescribed anxiolytic drugs acting through GABA receptors^38^. In rodents, administration of GABA_A_R agonists, including muscimol, also decreases the anxiety level^45,46^. GABA is synthesized from glutamate, which is transported into cells by membrane proteins. It is worth to note that four sodium-dependent amino acid transporters were dysregulated in the mutants. Whereas expression of the *Slc38a3* and *Slc38a5* were reduced, *Slc38a1* and *Slc38a2* expression were increased. These alterations were consistent with reported anxiety phenotype. For example, *Slc38a1* was overexpressed in *MeCP2* mutants, a mouse models for RETT syndrome with communication deficit, motor impairments, hand stereotypies^47^, and anxiety^48^. *Slc38a3* is an anxiety-related response locus *QTL15* in rat. *Slc38a3* and *Gng4* expression were altered in *EFhd2* knockout mouse associated with anxiety and alcohol addiction^49^. *Slc38a5* was one of the two genes dysregulated in *Rai1*^*+/−*^ mice with epilepsy, mental retardation, and anxiety disorder^50^.

There are >12 subtypes of GABA interneurons in mammalian brain^21^. Transcriptome analyses in this report showed significant reduction of many GABA interneuron subtypes in the *Ulk4* hypomorphs, including Pvalb, Sst, Cck, Npy, and Nos3, which constituted three quarters of total GABA interneurons in normal mice. Parvalbumin, the most abundant subtype whose deficiency is a fundamental pathology in neurodevelopmental disorders^22,23^, is significantly downregulated in *Ulk4* mutants.

Alterations in the density and number of GABAergic neurons in the brain occur throughout postnatal development^51–53^. It is not possible to identify exactly when *Ulk4* deletion would specifically alter GABAergic cell density and/or function but rather it is likely that alterations occur along the neurodevelopmental pathway. Despite this, RNA sequencing was done on P12 *Ulk4*^*tm1a/tm1a*^ and WT mice which reveal alterations in expression of GABA neuronal subtypes, and receptor subtypes. Furthermore, in 2-month old, we show again regional reduction of Gad67^+^ neurons in 2-month mice, the time correlating with behavioral testing.

GABA interneurons play their inhibition roles largely via brain GABA_A_Rs and GABA_B_Rs. The fast short-lasting “phasic” inhibition is typically generated by the activation of postsynaptic GABA_A_Rs following action potentials in presynaptic interneurons, and the “tonic” extra-synaptic inhibition is activated by ambient GABA in the extracellular space through molecularly and functionally specialized GABA_A_Rs. Remarkably the postsynaptic GABAergic signaling was also disrupted in *Ulk4* mutants, and this included significant upregulation of GABA_A_R subunits *Gabra1*, *Gabra3*, *Gabra4*, *Gabra5*, and *Gabrb3*. Changes in these receptor subunits are consistent with the anxiety phenotype. For example, the human *GABRA5* and *GABRB3* are located in the 15q11.2-q13 region, and maternal duplications of 15q11.2-q13 leads to neurodevelopmental disorders including ASD, and their clinical symptoms often include anxiety, emotional lability, tantrums, and hyperactivity^54^. Although deletion of *Gabra1* in amygdala did not affect anxiety behavior^55^, Gabra3-mediated tonic inhibition in the amygdala was essential in regulating fear and anxiety. The Gabra3-selective benzodiazepine site agonist and anxiolytic compound TP003 increased tonic currents and markedly dampen excitability in BLA principal cells^56^. Gabra4 was involved in fear extinction learning, and knockout of the extrasynaptic GABA_A_Rs facilitated fear extinction^57^. Altered expression also included *Nsf*, which was shown to downregulate GABA_A_Rs with PKCε^58^. We, however, cannot rule out that *Ulk4* deletion may affect GABAergic neurotransmission in other brain regions, and Ulk4 may be also expressed in other neurons such as glutamatergic neurons. Examination of effects in other neurons will be the focus of follow-up studies.

Sex bias is common in brain disorders and sex is known to influence GABAergic transmission and anxiety-related behavior^59^. The data herein demonstrate that *Ulk4* deletion alters various GABAergic parameters; however, assessment of such parameters was not conducted in a sufficient number of males and females separately to enable sex differences to be analyzed at this level. Despite subtle sex-related differences are observed in different behavioral paradigms, the strongest phenotype is the increased anxiety from plus maze test, which are consistent in both male and female mutants. Thus, the data suggest that *Ulk4* deletion modulates brain development and results in GABAergic changes which may underlie the anxiety-related phenotype.

In summary, we have shown for the first time that *Ulk4* haploinsufficiency in mice leads to increased anxiety-related behavior with disturbed GABAergic signaling. Ulk4 is involved in the maintenance of the excitation/inhibition balance, which is commonly disturbed in neurodevelopmental and neuropsychiatric disorders. Therefore, regulation of the ULK4 activity may present an alternative route of drug exploration for neurodevelopmental and neuropsychiatric illness.

## Electronic supplementary material

Supplemental Figure 1

Supplemental Figure S2

Supplemental Figure S3

Supplemental Figure S4
