# Supplementary figures and images for: Ulk4 regulates GABAergic signaling and anxiety-related behavior
Source: Transl Psychiatry. 2018 Feb 2;8:43. doi: 10.1038/s41398-017-0091-5 (PMC5804027; doi:10.1038/s41398-017-0091-5)

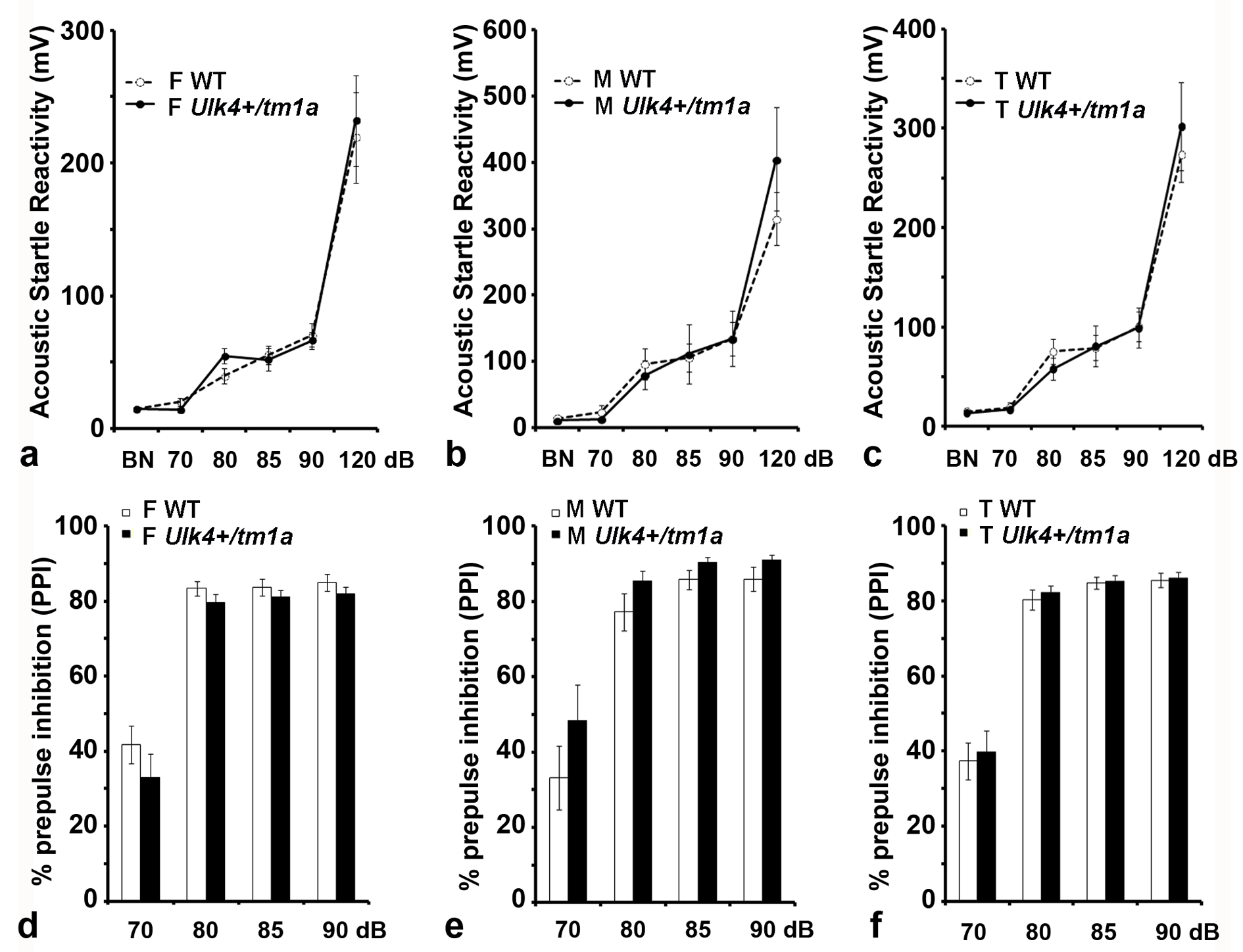

Supplement: Supplementary file 1 — Supplemental Figure 1 [file 41398_2017_91_MOESM1_ESM.tif]

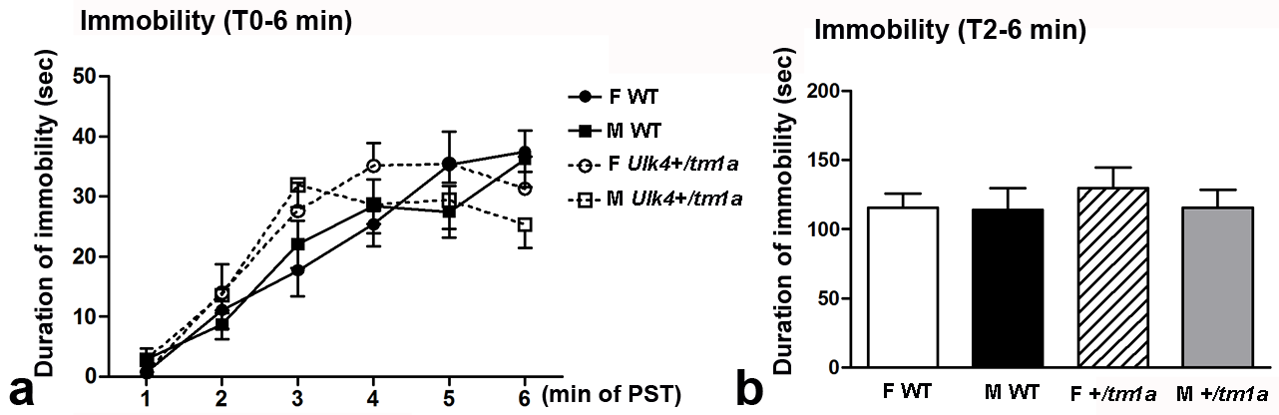

Supplement: Supplementary file 2 — Supplemental Figure S2 [file 41398_2017_91_MOESM2_ESM.tif]

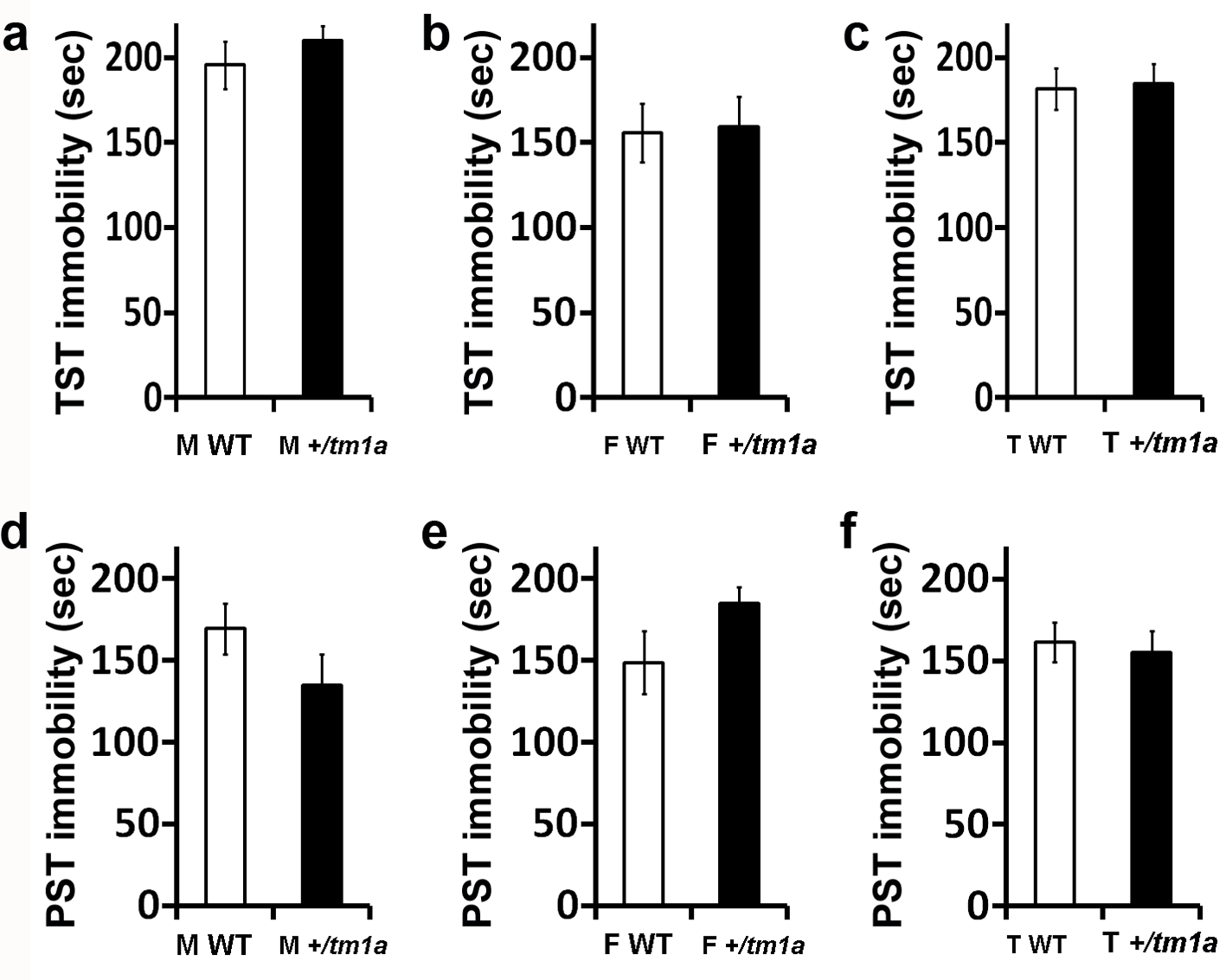

Supplement: Supplementary file 3 — Supplemental Figure S3 [file 41398_2017_91_MOESM3_ESM.tif]

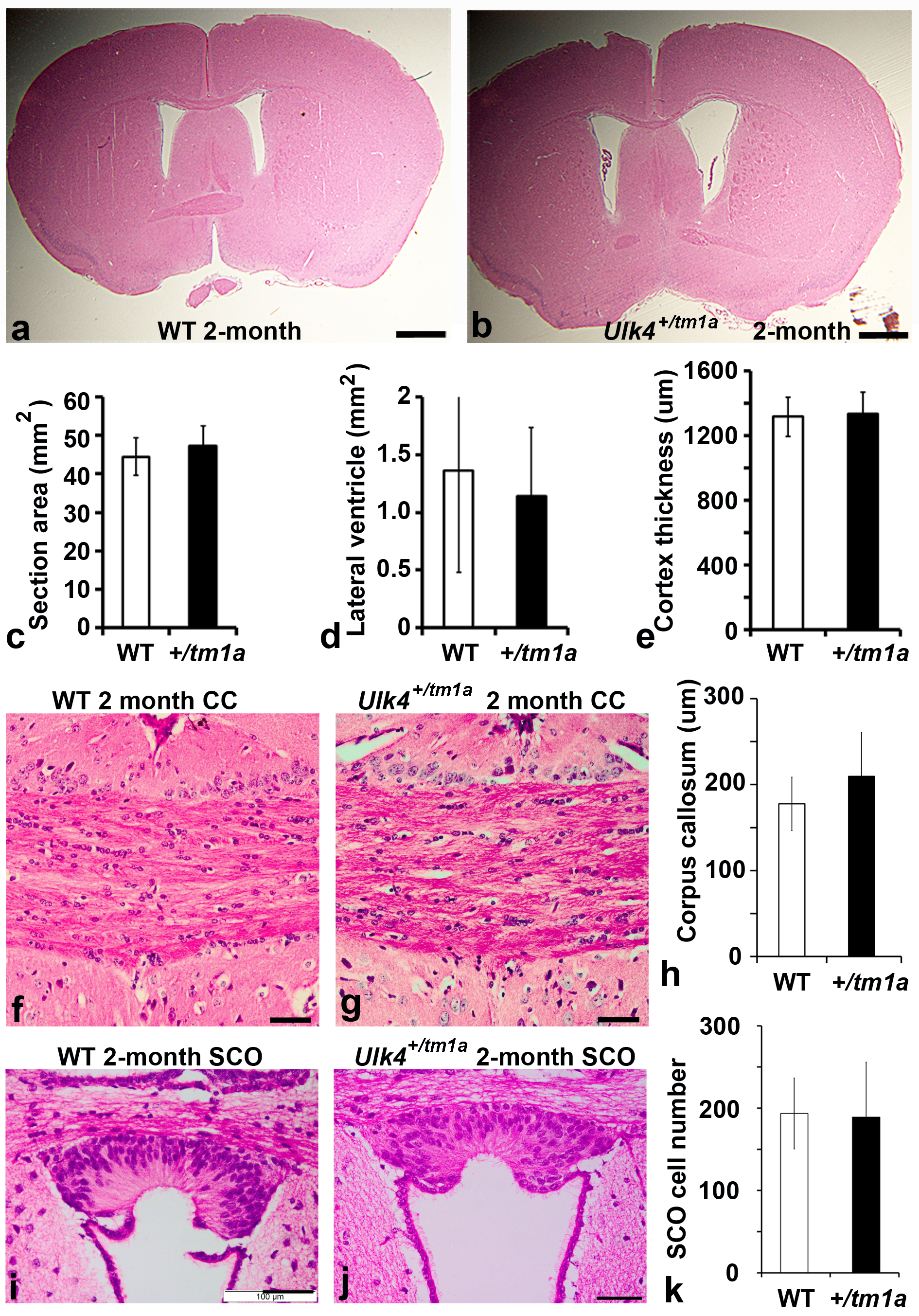

Supplement: Supplementary file 4 — Supplemental Figure S4 [file 41398_2017_91_MOESM4_ESM.tif]
